# Digital Home Learning Activities in Early Childhood: Associations with Socio-Emotional Difficulties

**DOI:** 10.3390/bs16050740

**Published:** 2026-05-09

**Authors:** Katerina Krousorati, Athanasios Gregoriadis, Anastasia Vatou

**Affiliations:** 1 Department of Early Childhood Education and Care, International Hellenic University, 57400 Thessaloniki, Greece; anastasia.vatou@ihu.gr; 2 School of Early Childhood Education and Care, Aristotle University of Thessaloniki, 54124 Thessaloniki, Greece; asis@nured.auth.gr

**Keywords:** digital home learning activities, Home Learning Environment, Home Learning Environment Questionnaire, home learning ecosystem, socio-emotional difficulties, early childhood

## Abstract

Families play a central role in shaping young children’s learning and development through everyday interactions and shared activities in the home environment. As digital technologies have become increasingly embedded in family life, digital home learning activities have emerged as a meaningful dimension of the broader Home Learning Environment, although research in this area remains limited. The present study examined the psychometric structure and characteristics of digital home learning activities and explored their associations with preschool children’s socio-emotional difficulties, as well as demographic factors related to variation in these activities across families. Data were collected from two independent parent samples (*N* = 308) of families with preschool-aged children. Digital home learning activities were assessed using the digital home learning activities subscale of the Home Learning Environment Questionnaire, while children’s socio-emotional difficulties were measured using the Strengths and Difficulties Questionnaire (Group A) and selected items from the Child Behavior Checklist (Group B). Confirmatory factor analyses supported a unidimensional structure with satisfactory reliability across both samples. Hierarchical regression analyses showed that digital home learning activities were generally not associated with children’s socio-emotional difficulties after demographic characteristics were taken into account. However, parent age and parent education were associated with variation in these activities across families.

## 1. Introduction

Early childhood is a foundational period of development, during which children’s learning is shaped not only by formal early education settings but also by everyday experiences within the family context (Bornstein, 2019; Gregoriadis & Evangelou, 2022). Developmental and ecological perspectives have long emphasized that routine interactions, play, and shared activities in the home provide essential opportunities through which young children acquire early competencies and socio-emotional skills (Bronfenbrenner, 1979; Kerr, 2019; Navarro & Tudge, 2023). This combination of learning-related activities, interactions, and resources provided by family members is commonly conceptualized as the Home Learning Environment (HLE) (Tamis-LeMonda et al., 2019), which has been consistently associated with children’s cognitive and academic development as well as with aspects of socio-emotional functioning (Hall et al., 2019; Li et al., 2023; Rose et al., 2018; Sammons et al., 2015).

In recent years, digital technologies have become increasingly embedded in children’s everyday home activities. For many young children, digital media are first encountered in family settings, where caregivers play an important role in shaping how, when, and for what purposes digital tools are used, including through mediation, guidance, and shared engagement (Chaudron et al., 2018). As a result, digital home learning activities have become a meaningful dimension of the broader HLE. However, research in this area remains fragmented. Much of the existing literature has focused either on children’s overall digital media exposure (e.g., Radesky & Christakis, 2016; Kabali et al., 2015) or on specific educational technologies and apps (e.g., Griffith & Arnold, 2019; Neumann, 2018; Palaiologou, 2016; Papadakis et al., 2019), whereas fewer studies have examined digital home learning activities within the broader ecological context of family learning processes (Gregoriadis & Evangelou, 2022; Krousorati et al., 2022). Moreover, findings regarding the relation between children’s digital experiences at home and socio-emotional functioning remain limited and mixed, with some studies pointing to potential risks and others highlighting the importance of context, co-use, and caregiver mediation (e.g., Konok et al., 2024; Lehrl et al., 2021).

Based on the previous arguments, the present study examines digital home learning activities in families with preschool-aged children. Specifically, it investigates their psychometric structure and descriptive characteristics across two independent parent samples, examines their associations with children’s socio-emotional difficulties and explores the demographic factors related to variation in these activities across families.

### 1.1. Literature Review and Theoretical Framework

#### 1.1.1. The Home Learning Environment and Digital Home Learning Activities

The Home Learning Environment (HLE) is a multidimensional construct that encompasses the various ways in which families support children’s early learning and development. It generally refers to the learning opportunities, resources, and interactions available within the family context that foster children’s development outside formal educational settings (Toth et al., 2019). The HLE is commonly understood to include three interrelated components: the physical home environment and the availability of learning materials, the learning activities that take place both inside and outside the home, and parent–child interactions during engagement in these activities (Tamis-LeMonda et al., 2019). Each of these components contributes to children’s learning and development beyond the classroom (Sammons et al., 2015).

Research on the HLE has followed two main approaches to its conceptualization and operationalization. The first adopts a holistic perspective, viewing the HLE as a broad context that reflects the overall quality and quantity of learning stimulation available within the family microsystem (Dearing & Tang, 2010). From this perspective, diverse learning experiences and interactions are combined into a general indicator of support for learning at home (Kluczniok et al., 2013). The second approach conceptualizes the HLE in domain-specific terms, focusing on particular content areas through which learning is supported. This perspective has led to the examination of dimensions such as the home literacy environment (Rose et al., 2018), the home numeracy environment (Cosso et al., 2024), and, more recently, the home science environment (Junge et al., 2021; Zhang et al., 2019) and the digital dimension of the HLE (Bonanati & Buhl, 2022; Lehrl et al., 2021; Sonnenschein et al., 2021). Although both approaches have advanced understanding of family-based learning, each also has limitations: domain-specific approaches may overlook the cumulative and interacting influences that shape children’s development, whereas broader approaches may not adequately capture domain-relevant processes (Gregoriadis & Evangelou, 2022; Wirth et al., 2023). The Home Learning Ecosystem framework is particularly useful in this regard because it bridges holistic and domain-specific perspectives by conceptualizing home learning practices as embedded in broader family learning systems (Gregoriadis & Evangelou, 2022).

The present study draws on the Home Learning Ecosystem framework, which offers an integrative perspective on learning experiences in the family context (Gregoriadis & Evangelou, 2022). Within this framework, digital home learning activities are understood as embedded within the broader HLE rather than as isolated indicators of children’s digital exposure. From this perspective, digital learning opportunities are shaped by everyday family routines and by the ways in which parents and children engage in shared digital learning activities at home. The Home Learning Ecosystem framework is therefore particularly relevant to the present study because it provides a theoretical basis for examining digital home learning activities as a distinct, yet integrated, dimension of the broader HLE.

The growing presence of digital technologies in family life has added an important digital dimension to young children’s home learning experiences (Kucker, 2021). Evidence suggests that young children are introduced to screen-based media at increasingly younger ages and that digital technologies have become part of everyday home life for many families (Dumuid, 2020; Kabali et al., 2015; Palaiologou, 2016; Papadakis et al., 2019). Many preschool-aged children begin developing digital skills at home before entering school, making parents and caregivers central in shaping children’s opportunities for digital literacy and digital engagement (Chaudron et al., 2018; Niklas et al., 2025). In this regard, parental mediation and scaffolding have been highlighted as important mechanisms through which children’s digital experiences are organized and supported within the home context (Gregoriadis & Evangelou, 2022).

As digital technologies have become increasingly embedded in family life, researchers have begun to examine how digital tools and practices contribute to children’s learning experiences at home. Related work has referred to the digital home learning environment and to digital home learning activities in order to describe the opportunities and practices through which children engage with digital technologies in the home context (Bonifacci et al., 2026; Krousorati et al., 2022; Lehrl et al., 2021; Segers & Kleemans, 2020). In the present study, however, the focus is specifically on digital home learning activities, that is, shared parent–child digital activities for learning purposes within the home environment (Gregoriadis & Evangelou, 2022). This distinction is important because broader discussions of children’s digital lives often include device access, screen time, or general media exposure (Xu & Qiao, 2025) whereas digital home learning activities refer more specifically to learning-oriented digital practices in which parents and children engage together.

Discussions of children’s digital media use have often focused on whether digital engagement displaces more traditional home learning experiences. An alternative perspective suggests that digital technologies may complement, rather than replace, other forms of home learning (Bonifacci et al., 2026). In practice, digital learning at home may begin early through children’s observation and imitation of the digital practices of parents and older siblings (Livingstone et al., 2015) and may also take the form of shared caregiver–child activities, such as using educational apps, engaging with digital books, or jointly exploring online content. This highlights the importance of understanding digital home learning activities not merely as indicators of digital exposure, but as learning-related practices embedded in everyday family interactions.

Emerging evidence further suggests that different forms of digital engagement may vary in their developmental significance. For example, passive forms of media use may differ from more interactive and shared forms of engagement in the extent to which they elicit parent–child interaction and support learning opportunities (Connell et al., 2015; Hasanen et al., 2021). Despite growing interest in children’s digital experiences, the digital dimension of the HLE remains less well understood than more established domains such as literacy and numeracy. In particular, relatively few studies have examined digital home learning activities as a distinct dimension of the broader HLE and considered how these activities are structured within families of preschool-aged children (Bonanati & Buhl, 2022; Lehrl et al., 2021).

Taken together, the available literature suggests that digital home learning activities should be understood as a meaningful dimension of the broader HLE rather than as simple indicators of children’s digital exposure. In addition, compared with more established dimensions of the HLE, such as home literacy and home numeracy, digital home learning activities remain conceptually and empirically less developed. More specifically, limited attention has been paid to how these activities are structured within families of preschool-aged children and whether they can be meaningfully examined as a distinct dimension of the HLE. This highlights the need for research that clarifies the characteristics and psychometric structure of digital home learning activities within an integrated theoretical framework.

#### 1.1.2. Digital Home Learning Activities and Children’s Socio-Emotional Difficulties

A substantial body of research has documented the importance of the HLE in infancy (Hall et al., 2019) and early childhood (Toth et al., 2019), demonstrating its associations with children’s developmental functioning, cognitive competencies (Bonifacci et al., 2022; Tamis-LeMonda et al., 2019), mental health and wellbeing (Lee & Xie, 2018), later academic achievement (Niklas & Schneider, 2017; Wang, 2025), and learning-related outcomes such as literacy (Segers & Kleemans, 2020), numeracy (Cosso et al., 2024), and early science knowledge (Junge et al., 2021; Zhang et al., 2019). Associations between the HLE and children’s social, emotional, and behavioural development have also been reported (Li et al., 2023; Rose et al., 2018; Wirth et al., 2020), although this area has received comparatively less attention than research on cognitive and academic outcomes.

Within this broader literature, evidence on digital home learning activities and children’s socio-emotional functioning remains limited. One of the few studies directly examining analog and digital dimensions of the HLE found that, among preschool-aged children, more frequent digital HLE activities were associated with weaker socio-emotional skills, whereas analog HLE activities showed more favourable associations; at the same time, digital activities were positively associated with academic outcomes (Lehrl et al., 2021). Related evidence from the broader literature on young children’s digital experiences at home also points to mixed associations with socio-emotional functioning. For example, screen exposure has been associated with children’s behavioural outcomes, although such work does not assess digital home learning activities as a distinct construct (Bonifacci et al., 2026). Similarly, broader evidence on young children’s digital technology use has reported small negative associations with psychosocial wellbeing, while other studies have linked higher digital screen exposure with more emotional symptoms and highlighted the role of the parent–child relationship in these associations (Harverson et al., 2025; Xu & Qiao, 2025). Another study found that frequent use of mobile devices to calm children aged 3–5 was associated with greater emotional reactivity and poorer executive functioning. Longitudinally, only emotional reactivity showed a bidirectional link with device use for calming, with stronger effects in boys and children higher in surgency (Radesky et al., 2023). These studies are informative, but they address broader forms of digital exposure and engagement and their associations with children’s socio-emotional outcomes rather than digital home learning activities specifically. The limited evidence concerning associations between digital home learning activities and children’s socio-emotional and behavioural difficulties supports the need for further research in this area.

At the same time, some evidence suggests that the developmental significance of children’s digital experiences may depend on how these experiences are structured and supported within the family context. Reviews of young children’s home digital literacy practices have emphasized the importance of parental mediation, while studies of joint media engagement have shown that shared parent–child digital practices may be more relevant for social development than screen time alone (Chen & Yeung, 2026; Soyoof et al., 2024). Taken together, these findings suggest that the developmental significance of children’s digital experiences at home may differ according to the nature of the activities in which parents and children engage together. In the present study, digital home learning activities are therefore operationalized as shared, learning-oriented practices rather than as overall screen exposure. Given the mixed evidence reported in preschool samples and the distinction between learning-oriented co-use and broader digital exposure (e.g., Bonifacci et al., 2026; Lehrl et al., 2021; Radesky et al., 2023), associations with socio-emotional and behavioural difficulties may not be uniform across domains. This highlights the need for further research examining whether, and to what extent, parent-reported digital home learning activities are associated with preschool children’s socio-emotional and behavioural difficulties.

#### 1.1.3. Demographic Factors Associated with Digital Home Learning Activities

Understanding how digital home learning activities vary across families is an important complement to research examining their associations with children’s socio-emotional outcomes. A demographic perspective helps situate digital home learning activities within the broader family context, acknowledging that opportunities for shared digital learning may be shaped by characteristics of the child and the parent (Paulus et al., 2024). This is also consistent with the Home Learning Ecosystem perspective, which conceptualizes children’s learning experiences as embedded in everyday family routines rather than as uniform exposures (Gregoriadis & Evangelou, 2022).

Existing evidence on demographic correlates of digital home learning activities remains limited. However, available studies suggest that child age is likely to be relevant, as digital HLE activities appear to increase across early childhood (Paulus et al., 2024). In Lehrl et al. (2021), shared digital HLE activities increased with age, indicating that digital learning practices become more common as children move from toddlerhood into the preschool years. Evidence regarding child sex is less consistent. Paulus et al. (2024) found no significant gender differences, while Bonanati and Buhl (2022) observed small gender differences and Hasanen et al. (2021) found that male child gender was associated with more parental co-participation in children’s digital media use, although this study addressed broader co-participation in digital media use rather than digital home learning activities specifically. Taken together, these findings suggest that child age and, to a lesser extent, child sex may be relevant demographic correlates to consider when examining variation in digital home learning activities.

Parent characteristics may also be relevant, although the available evidence is stronger for broader parental mediation and co-participation practices than for digital home learning activities specifically. Research on parental mediation has shown that parental education is associated with differences in how parents guide children’s media use, with lower-educated parents more often reporting difficulties or relying on more restrictive approaches to mediation (Nikken & Opree, 2018; Paulus et al., 2024). Related work has also shown that lower parental age is associated with more parental co-participation in children’s digital media use (Hasanen et al., 2021). The above findings highlight the need for further research examining which demographic characteristics are associated with parent-reported digital home learning activities in families with preschool-aged children. In the present study, parent education and parent age were therefore included as demographic indicators that may help account for variation in digital home learning activities across families.

### 1.2. The Present Study

The first research gap concerns the limited examination of digital home learning activities within the broader HLE. Although research on young children’s engagement with digital media is growing (e.g., Sonnenschein et al., 2021; Soyoof et al., 2024), the digital dimension of the HLE and the home learning activities remains underexamined. Most existing research has focused on more traditional HLE practices, such as shared reading and play-based learning, providing limited insight into how digital home learning activities are structured within families of preschool-aged children (e.g., Bonanati & Buhl, 2022; Lehrl et al., 2021). This gap constrains current theoretical understanding and limits the extent to which digital home learning activities can be conceptualized as a meaningful dimension of early learning within the broader HLE (Gregoriadis & Evangelou, 2022).

A second research gap concerns the limited evidence on the association between digital home learning activities and children’s socio-emotional and behavioural difficulties. Although a substantial body of research has examined associations between the HLE and children’s competencies in areas such as early literacy, numeracy, cognitive development, and later school achievement, evidence regarding socio-emotional outcomes remains comparatively limited, particularly with respect to the digital dimension of the HLE. Direct evidence specifically addressing digital home learning activities and children’s socio-emotional functioning is still scarce (Lehrl et al., 2021), and broader related evidence from studies of young children’s digital experiences at home has yielded mixed findings (Bonifacci et al., 2026; Xu & Qiao, 2025). Further empirical work is therefore needed to clarify whether, and to what extent, parent-reported digital home learning activities are associated with children’s emotional and behavioural functioning after accounting for demographic characteristics.

A third research gap concerns the limited understanding of the demographic factors associated with digital home learning activities. Although previous research has identified demographic differences in children’s general digital media use, much less is known about how key characteristics, such as child age, child sex, and parent education, are related specifically to engagement in digital home learning activities during the preschool years (Hasanen et al., 2021; Lehrl et al., 2021; Paulus et al., 2024). In addition, digital home learning activities have rarely been examined as a distinct dimension of the broader HLE in relation to demographic variation, limiting current understanding of how such activities may be distributed across families. Addressing this gap is important for clarifying the social patterning of digital home learning activities and for informing efforts to support more equitable digital learning opportunities in early childhood.

To address these gaps, the present study examined the characteristics and psychometric structure of digital home learning activities and investigated their associations with preschool children’s socio-emotional and behavioural difficulties. Using two independent parent samples, each of which completed the digital home learning activities subscale of the HLEQ alongside either the Strengths and Difficulties Questionnaire (SDQ) or the Child Behavior Checklist (CBCL), the study was guided by the following research questions:

RQ1: What are the characteristics of digital home learning activities, and what is their psychometric structure across two parent samples?
RQ2: What is the association between digital home learning activities and preschool children’s socio-emotional and behavioural difficulties, as measured by the SDQ and the CBCL, after accounting for demographic characteristics?
RQ3: Which demographic factors are associated with digital home learning activities (e.g., child age, child sex, parent education, parent age)?

Based on prior literature, the present study formulated three main expectations. First, the digital home learning activities subscale was expected to demonstrate a unidimensional structure and satisfactory reliability across the two independent samples (RQ1). Second, digital home learning activities were expected to be associated with children’s socio-emotional difficulties, although prior findings suggested that these associations might be weak or non-significant (RQ2). Third, digital home learning activities were expected to vary according to child and family background characteristics, particularly parent education and other demographic factors (RQ3).

## 2. Materials and Methods

### 2.1. Participants and Procedure

This cross-sectional study used convenience sampling to recruit two independent samples of parents of preschool-aged children. In both samples, the same digital home learning activities subscale of the HLEQ was administered alongside different measures of socio-emotional and behavioural difficulties (SDQ in Group A and CBCL in Group B). The digital home learning activities subscale was administered with the same items, response format, and scoring procedure across groups, supporting comparability in the assessment of digital home learning activities. Its psychometric structure was also examined separately in each group. Administering one socio-emotional instrument per sample reduced respondent burden while allowing the study aims to be examined across two established assessment frameworks. The use of two widely used parent-report measures also enabled consideration of whether patterns of association with digital home learning activities were broadly similar across instruments. Accordingly, outcome analyses were conducted separately within each sample, and results were interpreted within each instrument rather than as direct comparisons of SDQ and CBCL scores.

The total sample comprised 308 parents. Group A consisted of 133 parents and was used for analyses involving the SDQ, whereas Group B consisted of 175 parents and was used for analyses involving the CBCL. In Group A, the mean age of the parents was 36.4 years (*SD* = 5.8), and the mean age of their children was 35.3 months (*SD* = 10.2). In Group B, the mean age of the parents was 36.8 years (*SD* = 4.8), and the mean age of their children was 36.1 months (*SD* = 3.6). Demographic characteristics of the two samples are presented in Table 1.

Data were collected during the 2022–2023 and 2023–2024 school years. Both groups were recruited through preschool settings using comparable convenience-sampling and parent-contact procedures during the broader data collection period. Parents were contacted through preschools and received written information about the aims and procedures of the study, together with an invitation to participate. Consent forms and invitation letters were then distributed to parents. Questionnaires, in both paper-and-pencil and online formats, were distributed either through educators or by email and were completed anonymously. Participants were informed that they could withdraw from the study at any time without providing a reason. No identifying information was collected, and no incentives were provided for participation. Data were used solely for research purposes and were stored securely. The study protocol was reviewed and approved by the Ethics Committee of the authors’ department (license approval protocol code EA230235).

### 2.2. Measures

#### 2.2.1. Digital Home Learning Activities

Digital home learning activities were assessed using the digital home learning activities subscale of the Home Learning Environment Questionnaire (HLEQ; Krousorati et al., 2022), developed within the Home Learning Ecosystem framework (Gregoriadis & Evangelou, 2022). The subscale includes five items assessing the frequency of shared parent–child digital learning activities, such as shared e-book reading, joint use of educational apps, and joint use of the internet for learning purposes. Responses were provided on a 6-point Likert-type scale ranging from 1 (“never”) to 6 (“always”). Item scores were averaged to form a composite score, with higher scores indicating more frequent engagement in digital home learning activities. Previous research has supported the psychometric adequacy of the subscale (Krousorati et al., 2022). In the present study, internal consistency was good in both samples (McDonald’s *ω* = 0.894 for Group A [SDQ sample] and McDonald’s *ω* = 0.837 for Group B [CBCL sample]).

#### 2.2.2. Strengths and Difficulties Questionnaire

Children’s socio-emotional and behavioural functioning in the first sample (Group A) was assessed using the parent-report version of the Strengths and Difficulties Questionnaire for children aged 2–4 years (SDQ; Goodman, 1997). The SDQ is a widely used behavioural screening instrument comprising 25 items rated on a 3-point scale (0 = not true, 1 = somewhat true, 2 = certainly true). The measure yields five subscales: Emotional Symptoms, Conduct Problems, Hyperactivity/Inattention, Peer Problems, and Prosocial Behaviour. Following standard scoring procedures, positively worded items contributing to difficulty scales were reverse-scored. Higher scores on the four difficulty subscales indicate greater difficulties, whereas higher scores on the Prosocial Behaviour subscale indicate stronger prosocial skills.

In addition to the five subscales, a Total Difficulties score was calculated by combining the Emotional Symptoms, Conduct Problems, Hyperactivity/Inattention, and Peer Problems subscales. Broader indicators of internalizing difficulties and externalizing difficulties were also computed. Internalizing difficulties were indexed by combining Emotional Symptoms and Peer Problems, whereas externalizing difficulties were indexed by combining Conduct Problems and Hyperactivity/Inattention.

The SDQ has been used and validated in Greek samples and has shown adequate psychometric properties in previous research (Bibou-Nakou et al., 2019; Giannakopoulos et al., 2009, 2013; Gomez et al., 2021; Kotsis et al., 2024; Tsakalidis et al., 2023; Vatou, 2023; Vourdas et al., 2018). In the present study, analyses focused on the SDQ Total Difficulties score and the composite indices of Internalizing Difficulties and Externalizing Difficulties, which were used as the primary SDQ outcomes. Internal consistency was satisfactory for the Total Difficulties score (McDonald’s *ω* = 0.800), Internalizing Difficulties (*ω* = 0.757) and Externalizing Difficulties (*ω* = 0.700).

#### 2.2.3. Child Behavior Checklist

Children’s socio-emotional and behavioural difficulties in the second sample (Group B) were assessed using selected items from the Greek version of the Child Behavior Checklist for ages 1½–5 (CBCL/1½–5; Achenbach & Rescorla, 2000; Tsaousis, 2009). The CBCL/1½–5 is a widely used caregiver-report instrument that assesses a broad range of emotional and behavioural problems in preschool-aged children on a 3-point scale (0 = not true, 1 = somewhat or sometimes true, 2 = very true or often true). The CBCL/1½–5 has demonstrated satisfactory psychometric properties in Greek preschool samples (e.g., Roussos et al., 1999; Kadoglou et al., 2024; Vatou et al., 2025).

Consistent with previous Greek research using the same selected CBCL/1½–5 items (Vatou et al., 2025), the present study derived two composite scores indexing broader internalizing and externalizing difficulties. The internalizing composite included items reflecting anxious/depressed and withdrawn behaviours, whereas the externalizing composite included items reflecting attention problems and aggressive behaviours. The two composite scores were computed by summing the relevant items, with higher scores indicating greater socio-emotional and behavioural difficulties. Internal consistency in the present sample was good for both composites (McDonald’s *ω* = 0.746 for Internalizing Problems and McDonald’s *ω* = 0.861 for Externalizing Problems).

#### 2.2.4. Indicators of Child and Family Background

Parents provided information on child and family background characteristics. Child-related variables included child age (in months) and child sex (boy/girl). Parent-related variables included parent age (in years) and parent education. Parent education was recorded as the highest level of formal education completed. These variables were used as demographic indicators in subsequent analyses to account for variation across participants.

### 2.3. Statistical Analysis

All statistical analyses were conducted using JAMOVI (version 2.6.2) (The Jamovi Project, 2025). Analyses were performed separately for the two parent samples when socio-emotional outcomes were assessed with different instruments (SDQ in Group A; CBCL in Group B), whereas analyses addressing RQ3 were conducted on the combined dataset. Because the SDQ and CBCL yield outcomes on different metrics, analyses examining associations between digital home learning activities and socio-emotional and behavioural difficulties were conducted within each sample. Cross-sample interpretation was therefore limited to convergent patterns rather than direct between-group contrasts. Preliminary analyses included descriptive statistics for all study variables and inspection of distributional properties.

To address RQ1, descriptive statistics were computed for the digital home learning activities composite score and its constituent items. Confirmatory factor analyses (CFA) were conducted separately in each parent sample to examine the psychometric structure of the digital home learning activities subscale. In line with the Home Learning Ecosystem framework (Gregoriadis & Evangelou, 2022) and prior empirical findings (Krousorati et al., 2022), a one-factor model was specified a priori. CFA was selected to evaluate whether this theoretically derived unidimensional structure was supported in two independent samples. Model fit was assessed using multiple indices, including the chi-square statistic and its degrees of freedom, the Comparative Fit Index (CFI), the Root Mean Square Error of Approximation (RMSEA), and the Standardized Root Mean Square Residual (SRMR). Following Kline (2023), values close to 0.95 for CFI, 0.08 for SRMR, and 0.06 for RMSEA were taken to indicate good model fit. Where relevant, modification indices were inspected, and model adjustments were considered only when theoretically justifiable and substantively meaningful. Internal consistency was examined using McDonald’s *ω* (McDonald, 1999).

To address RQ2, hierarchical multiple linear regression models were estimated separately in each sample to examine the association between digital home learning activities and children’s socio-emotional and behavioural difficulties after accounting for demographic characteristics. In Group A, models were estimated for SDQ internalizing difficulties, externalizing difficulties, and total difficulties, whereas in Group B, models were estimated for CBCL internalizing and externalizing problems. In each model, child age, child sex, parent age, and parent education were entered at Step 1, and digital home learning activities were entered at Step 2. Preliminary bivariate associations were also examined using Spearman’s rank-order correlations (ρ).

Last, to address RQ3, a hierarchical multiple linear regression model was estimated in the combined sample to examine whether demographic characteristics were associated with digital home learning activities. Because preliminary analyses showed significant differences in digital home learning activities between Group A and Group B, sample group was entered as a control variable in the model. Thus, group, child age, child sex, parent age, and parent education were included as predictors, with digital home learning activities as the dependent variable. Model fit was evaluated using *R*^2^, adjusted *R*^2^, and the overall *F* test.

## 3. Results

### 3.1. Characteristics and Factor Structure of Digital Home Learning Activities

A confirmatory factor analysis (CFA) was conducted separately in each sample to examine the factor structure of the digital home learning activities subscale. In line with the original structure of the HLEQ, a one-factor model was specified, representing shared parent–child digital learning activities at home. Fit indices for the CFA models are presented in Table 2.

In Group A (*Ν* = 133), the initial CFA indicated mixed fit, χ^2^(5) = 19.30, *p* = 0.002, CFI = 0.969, SRMR = 0.028, RMSEA = 0.147, 90% CI [0.081, 0.210]. Inspection of the modification indices suggested allowing the residuals of the e-book reading item and the numeracy applications item to correlate. This modification was considered theoretically plausible because both items reflect specific learning-oriented digital activities and may therefore share residual variance beyond the general factor (item 7 and item 9). After allowing this residual covariance, model fit improved substantially, *χ*^2^(4) = 6.49, *p* = 0.165, CFI = 0.995, RMSEA = 0.068, SRMR = 0.019. Standardized factor loadings ranged from 0.547 to 0.969, and internal consistency was good (McDonald’s *ω* = 0.894).

In Group B (*Ν* = 175), the one-factor CFA showed good fit, *χ*^2^(5) = 7.43, *p* = 0.191, CFI = 0.993, RMSEA = 0.052, SRMR = 0.029. All standardized factor loadings were statistically significant and ranged from 0.524 to 0.922. Internal consistency was also good (McDonald’s *ω* = 0.837).

Descriptive statistics and standardized factor loadings for the digital home learning activities items are presented in Table 3. Across both samples, composite mean scores were below the midpoint of the response scale, indicating relatively infrequent engagement in shared digital home learning activities overall. More specifically, the mean composite score was higher in Group A (*M* = 2.74, *SD* = 1.14) than in Group B (*M* = 1.84, *SD* = 0.89). Because the two groups were independent samples and socio-emotional outcomes were assessed with different instruments, this descriptive difference was not interpreted as a direct between-group comparison of socio-emotional outcomes. The group difference in digital home learning activities was taken into account in the combined-sample analysis by including sample group as a control variable.

### 3.2. Associations Between Digital Home Learning Activities and Children’s Socio-Emotional and Behavioural Difficulties

Associations between digital home learning activities and children’s socio-emotional and behavioural difficulties were examined separately in the two samples, with SDQ outcomes analysed in Group A and CBCL outcomes analysed in Group B. To assess whether digital home learning activities were associated with these outcomes above and beyond demographic characteristics, hierarchical multiple regression analyses were conducted in each sample. In all models, child age, child sex, parent age, and parent education were entered in Model 1, and digital home learning activities were added in Model 2. Descriptive statistics and bivariate correlations are presented in Table 4 and Table 5, and the hierarchical regression models are reported in Table 6 and Table 7.

In the SDQ sample (Group A), digital home learning activities showed a small positive correlation with externalizing difficulties (ρ = 0.203, *p* < 0.05), but were not significantly correlated with internalizing difficulties (ρ = 0.058) or total difficulties (ρ = 0.156). As expected, strong positive correlations were observed among the SDQ indices, with externalizing difficulties strongly associated with total difficulties (ρ = 0.862, *p* < 0.001) and internalizing difficulties (ρ = 0.423, *p* < 0.001) (Table 4).

In the CBCL sample (Group B), digital home learning activities were not significantly correlated with either internalizing problems (ρ = 0.145) or externalizing problems (ρ = 0.058). A strong positive association was observed between CBCL internalizing and externalizing problems (ρ = 0.552, *p* < 0.001) (Table 5).

Results of the hierarchical regression analyses predicting SDQ outcomes are presented in Table 6. For SDQ internalizing difficulties, Model 1, which included demographic variables, was not significant (*p* > 0.05), *F*(5, 123) = 0.46, *R*^2^ = 0.018. Adding digital home learning activities in Model 2 did not significantly improve the model, Δ*R*^2^ = 0.004, Δ*F*(1, 122) = 0.55. Digital home learning activities were not a significant predictor of internalizing difficulties (*B* = 0.193, *SE* = 0.259, β = 0.071). For SDQ externalizing difficulties, Model 1 was significant, *F*(5, 124) = 2.97, *p* < 0.05, explaining 10.7% of the variance. Child sex emerged as a significant predictor (*B* = −1.259, *SE* = 0.496), with girls showing lower externalizing difficulties than boys. Model 2 remained significant, *F*(6, 123) = 3.10, *p* < 0.01, but the addition of digital home learning activities did not significantly improve model fit, Δ*R*^2^ = 0.025, Δ*F*(1, 123) = 3.47. Digital home learning activities were not a significant predictor of externalizing difficulties (*B* = 0.416, *SE* = 0.223, β = 0.166). For SDQ total difficulties, Model 1 was not significant, *F*(5, 120) = 1.17, *R*^2^ = 0.047. Adding digital home learning activities in Model 2 did not significantly increase explained variance (Δ*R*^2^ = 0.014, Δ*F* = 1.71). Digital home learning activities were not significantly associated with total difficulties (*B* = 0.540, *SE* = 0.414, β = 0.124).

Results for the CBCL outcomes are shown in Table 7. For CBCL internalizing problems, Model 1 including demographic variables was not significant, *F*(5, 168) = 1.02, *R*^2^ = 0.029. Adding digital home learning activities in Model 2 did not significantly improve the model (Δ*R*^2^ = 0.019, Δ*F* = 3.27). Digital home learning activities were not significantly associated with internalizing problems (*B* = 0.500, *SE* = 0.276, β = 0.139).

For CBCL externalizing problems, Model 1 was significant, *F*(5, 168) = 2.58, *p* < 0.05, explaining 7.1% of the variance. Child age emerged as a significant positive predictor (*B* = 3.869, *SE* = 1.615), while girls showed lower levels of externalizing problems than boys (*B* = −1.851, *SE* = 0.967). Model 2 remained significant, *F*(6, 167) = 2.35, *p* < 0.05, but the addition of digital home learning activities did not significantly improve model fit (Δ*R*^2^ = 0.007, Δ*F* = 1.19). Digital home learning activities were not significantly associated with externalizing problems (*B* = 0.593, *SE* = 0.544, β = 0.083).

### 3.3. Associations Between Digital Home Learning Activities and Demographic Factors

A hierarchical multiple regression analysis was conducted to examine whether demographic characteristics were associated with digital home learning activities in the combined sample. Preliminary analyses indicated that digital home learning activities differed significantly between the two samples, with Group A reporting higher levels than Group B. Therefore, sample group was included in the model as a control variable. The overall model was statistically significant, *F*(6, 300) = 13.60, *p* < 0.001, explaining 21.4% of the variance in digital home learning activities (*R*^2^ = 0.214, adjusted *R*^2^ = 0.198). Adding the demographic variables in Model 2 significantly improved model fit beyond group alone, Δ*R*^2^ = 0.048, Δ*F*(5, 300) = 3.69, *p* = 0.003.

In the final model, group remained a strong significant predictor, with participants in Group B reporting lower levels of digital home learning activities than those in Group A (*B* = −0.840, *SE* = 0.116, β = −0.764, *p* < 0.001). Parent age was negatively associated with digital home learning activities (*B* = −0.028, *SE* = 0.011, β = −0.134, *p* = 0.010). In addition, postgraduate education was associated with lower levels of digital home learning activities than secondary education (*B* = −0.447, *SE* = 0.179, β = −0.407, *p* = 0.013). Child age, child sex, and the contrast between bachelor/associate and secondary education were not significant predictors. No evidence of problematic multicollinearity was observed (see Table 8).

## 4. Discussion

This study investigated the structure and characteristics of digital home learning activities within the Home Learning Environment of preschool children utilizing two independent parent samples. Furthermore, this study examined the associations between these activities and children’s socio-emotional difficulties, as well as the demographic characteristics linked to variation in digital home learning activities across families. Three main findings emerged. First, digital home learning activities demonstrated a coherent and internally consistent construct across the two samples. Second, their associations with children’s socio-emotional difficulties were generally limited and did not remain statistically significant even after demographic characteristics were taken into account. Third, digital home learning activities were associated with selected parent characteristics, suggesting that they may vary across family contexts. Each of these findings is discussed below.

First, the results supported the expectation, showing that the digital home learning activities had a unidimensional structure and satisfactory reliability across the two independent samples. The analyses provided support for a one-factor structure, indicating that the examined digital home learning activities represent a coherent set of shared parent–child learning practices, that may constitute a distinct but integrated dimension of the broader Home Learning Environment. This finding is consistent with theoretical perspectives on the Home Learning Environment, which conceptualize learning opportunities in the home as clusters of related activities that are embedded in everyday parent–child interactions (Gregoriadis & Evangelou, 2022; Tamis-LeMonda et al., 2019), as well as with prior empirical findings (Krousorati et al., 2022). Whereas previous research has often focused on digital media exposure, digital device use or screen time (e.g., Chaudron et al., 2018; Kabali et al., 2015; Xu & Qiao, 2025), the present findings contribute to the literature by providing empirical support for the conceptualization of digital home learning activities as a coherent component of the Home Learning Environment (HLE) in families with preschool-aged children.

A further notable finding is that parents in both samples reported relatively low levels of engagement in digital home learning activities, suggesting that shared learning-oriented digital practices were not highly frequent in everyday family life. This pattern is broadly consistent with Lehrl et al. (2021), whose findings also suggested lower mean levels for digital than for analog home learning activities in preschool-aged children. It is also broadly in line with evidence suggesting that the digital dimension of the HLE is not a uniform feature of family life, but varies across specific forms of parental support and shared digital engagement (Bonanati & Buhl, 2022). Taken together, these findings suggest that, although digital home learning activities can be conceptualized as a coherent dimension of the HLE, they do not appear to constitute highly frequent everyday practices for many families with preschool-aged children.

Second, the findings indicated that digital home learning activities were generally not associated with children’s socio-emotional difficulties after demographic characteristics were taken into account. Although a small positive bivariate association with externalizing difficulties emerged in the SDQ sample, this association did not remain statistically significant in the adjusted models. This finding partly aligns with Lehrl et al. (2021), who found that more frequent digital HLE activities were associated with weaker socio-emotional skills in preschool-aged children. More broadly, however, the present findings differ from strands of the literature that have reported negative associations between children’s development and digital screen exposure, screen time, or the use of devices for calming and emotion regulation (e.g., Konok et al., 2024; Radesky et al., 2023; Xu & Qiao, 2025). One possible explanation is that these studies examined broader screen exposure or qualitatively different forms of digital engagement, whereas the present study focused specifically on shared, learning-oriented digital home learning activities, as theoretically positioned within the Home Learning Ecosystem framework (Gregoriadis & Evangelou, 2022). In addition, the outcomes examined here concerned socio-emotional difficulties rather than broader socio-emotional functioning. In this sense, the absence of significant associations may suggest that shared learning-oriented digital activities in the home are not linked to increased behavioural or emotional problems in early childhood. This may also imply that when parents engage with their children in these learning activities, they may use higher levels of guidance and mediation in contrast to when children simply are exposed and use screen or digital media devices (Chaudron et al., 2018). Thus, the developmental significance of children’s digital experiences may depend on how these experiences are structured within the family context, including whether they involve shared, guided, and learning-oriented engagement rather than passive or regulatory uses of digital media. This interpretation is also consistent with studies suggesting that shared digital media engagement and the quality of parent–child interaction may be more relevant for children’s social development than screen exposure alone (Chen & Yeung, 2026; Griffith & Arnold, 2019).

Third, the findings indicated that some demographic characteristics were associated with variation in digital home learning activities across families. In particular, parent age and parent education emerged as significant predictors of shared parent–child engagement in these activities. These results are broadly consistent with previous research suggesting that parent age and education may be related to differences in parental mediation, co-participation, and digital practices in the home context (Hasanen et al., 2021; Nikken & Opree, 2018; Paulus et al., 2024). More specifically, the present findings suggest that shared parent–child engagement in digital home learning activities may vary across families and may partly reflect differences in parental experiences, familiarity with digital resources, or preferences regarding the use of digital tools in children’s everyday learning activities. At the same time, the relatively modest amount of explained variance indicates that demographic characteristics alone do not fully account for variation in digital home learning activities, suggesting that other aspects of the family context, such as parental beliefs, mediation practices, or access to digital resources, may also play an important role.

Several limitations of the present study should be acknowledged. First, data were based on parent self-reports, which may be susceptible to subjective bias and social desirability effects and may not fully capture actual family practices. In addition, the digital home learning activities subscale was operationalized as a single composite across diverse digital learning activities, which may obscure activity-specific patterns. Future research could examine whether specific activities, such as shared e-book reading, educational applications, or web-based activities, show distinct associations with child outcomes. Second, children’s socio-emotional difficulties were assessed exclusively through parent questionnaires. Although widely used instruments were employed, future research could benefit from incorporating multiple assessment methods, such as direct observations, teacher reports, or standardized assessments, in order to obtain a more comprehensive evaluation of children’s socio-emotional and behavioural functioning. Third, the sample was not representative of Greek families, as the majority of participating parents had relatively high levels of education. As a result, the findings should be interpreted with caution when generalizing to more socioeconomically diverse populations. In addition, the cross-sectional design of the study does not allow conclusions about causal relationships between digital home learning activities and children’s development.

Future research should therefore examine the role of digital home learning activities using longitudinal designs and more diverse family samples. Further studies could also explore the quality of parent–child interactions during digital home learning activities and the ways in which parental mediation practices shape children’s digital experiences within the HLE.

From a practical perspective, the findings highlight the importance of supporting parents in engaging in shared, learning-oriented digital activities with their children. Providing guidance to families on how to integrate digital technologies into everyday learning interactions may help strengthen the broader HLE and support young children’s developmental experiences with digital media.

## 5. Conclusions

Children’s engagement with digital technologies begins in the early years. The present findings contribute to the literature on the digital dimension of the HLE by showing that digital home learning activities form a coherent set of shared parent–child practices that can be conceptualized as part of the broader HLE. These activities were not associated with increased socio-emotional difficulties, indicating that learning-oriented digital engagement within the family context does not appear to be linked to elevated behavioural or emotional problems in early childhood. Furthermore, engagement in digital home learning activities varied across families and was related to selected parent characteristics. Overall, the results underscore the importance of considering not only the amount of children’s digital media exposure, but also the nature and context of digital home learning activities within everyday family interactions when examining their developmental significance.

## Figures and Tables

**Table 1:** Demographic Characteristics of the Study Samples.

	Group A (*N* = 133) *M* (*SD*) or *n* (%)	Group B (*N* = 175) *M* (*SD*) or *n* (%)	Total (*N* = 308) *M* (*SD*) or *n* (%)
	Children		
Child sex			
Boys	70 (52.6%)	99 (56.6%)	169 (54.9%)
Girls	63 (47.4%)	76 (43.4%)	139 (45.1%)
Age of children (in months)	35.3 (*SD* = 10.2)	36.1 (*SD* = 3.6)	35.7 (*SD* = 7.2)
	Parents		
Age of parents (in years)	36.4 (*SD* = 5.8)	36.8 (*SD* = 4.8)	35.8 (*SD* = 5.4)
Parent’s educational level			
secondary education	35 (26.3%)	23 (13.1%)	58 (18.3%)
bachelor/associate degree	77 (57.9%)	102 (58.3%)	179 (58.1%)
post-graduate studies	21 (15.8%)	50 (28.6%)	71 (23.1%)

Note. Group A was used for analyses involving the SDQ, whereas Group B was used for analyses involving the CBCL. Both groups were combined only for analyses addressing RQ3.

**Table 2:** Confirmatory factor analysis results for digital home learning activities.

	*x* ^2^	df	CFI	RMSEA	SRMR
Group A	6.49	4	0.995	0.068	0.019
Group B	7.43	5	0.993	0.052	0.029

Note. CFI = Comparative Fit Index; RMSEA = Root Mean Square Error of Approximation; SRMR = Standardized Root Mean Square Residual.

**Table 3:** Descriptive statistics and standardized factor loadings for digital home learning activities items.

	Group A			Group B		
	(*N* = 133)			(*N* = 175)		
	Standardized Factor Loading	*M*	*SD*	Standardized Factor Loading	*M*	*SD*
The parent						
Shares reading an e-book with the child on smart devices (e.g., tablet, e-reader, smartphone)	0.859	2.81	1.43	0.597	1.66	1.06
Plays with the child with literacy applications (e.g., tablet, smartphone)	0.925	2.60	1.44	0.922	1.74	1.13
Plays with the child with numeracy applications (e.g., tablet, smartphone)	0.969	2.73	1.46	0.880	1.90	1.29
Surfs the web with the child (e.g., to find a story or a song)	0.660	2.00	1.19	0.582	2.46	1.40
Plays video games with the child	0.547	3.54	1.33	0.524	1.42	0.85
Digital home learning activities		2.74	1.14		1.84	0.89
McDonald’s *ω*	0.894			0.837		

**Table 4:** Descriptive statistics and bivariate correlations between digital home learning activities and SDQ outcomes in Group A (SDQ sample).

	*M* (*SD*)	1	2	3	4
1. Digital home learning activities	2.74 (1.14)	-			
2. SDQ Internalizing difficulties	12.9 (3.09)	0.058	-		
3. SDQ Externalizing difficulties	14.5 (2.87)	0.203 *	0.423 ***	-	
4. SDQ Total difficulties	27.3 (5.00)	0.156	0.797 ***	0.862 ***	-

Note. Entries are Spearman’s ρ coefficients. * *p* < 0.05, *** *p* < 0.001.

**Table 5:** Descriptive statistics and bivariate correlations between digital home learning activities and CBCL outcomes in Group B (CBCL sample).

	*M* (*SD*)	1	2	3
1. Digital home learning activities	1.84 (0.89)	-		
2. CBCL Internalizing problems	4.84 (3.20)	0.145	-	
3. CBCL Externalizing problems	11.6 (6.39)	0.058	0.552 ***	-

Note. Entries are Spearman’s ρ coefficients. *** *p* < 0.001.

**Table 6:** Hierarchical multiple regression analyses predicting SDQ outcomes from digital home learning activities in Group A.

	SDQ Internalizing Difficulties				SDQ Externalizing Difficulties				SDQ Total Difficulties			
	Model 1		Model 2		Model 1		Model 2		Model 1		Model 2	
Predictor	*B* (*SE*)	β	*B* (*SE*)	β	*B* (*SE*)	β	*B* (*SE*)	β	*B* (*SE*)	β	*B* (*SE*)	β
Child age (months)	−0.030 (0.028)	−0.099	−0.033 (0.028)	−0.107	−0.045 (0.025)	−0.158	−0.048 * (0.024)	−0.170	−0.079 (0.045)	−0.158	−0.085 (0.046)	−0.171
Child sex (0 = boy, 1 = girl)	−0.293 (0.561)	−0.095	−0.300 (0.562)	−0.097	−1.259 * (0.496)	−0.439	−1.258 * (0.492)	−0.439	−1.348 (0.907)	−0.270	−1.348 (0.904)	−0.270
Parent age (years)	0.041 (0.047)	0.077	0.050 (0.049)	0.095	−0.049 (0.042)	−0.100	−0.029 (0.042)	−0.060	−0.023 (0.076)	−0.027	0.004 (0.078)	0.005
Parent education (bachelor/associate vs. secondary education)	−0.070 (0.660)	−0.023	−0.068 (0.661)	−0.022	−0.172 (0.581)	0.060	−0.157 (0.575)	−0.055	−0.308 (1.069)	−0.062	−0.296 (1.066)	−0.059
Parent education (postgraduate vs. secondary education)	0.429 (0.882)	0.139	0.579 (0.907)	0.187	−1.314 (0.779)	−0.458	−0.969 (0.793)	−0.338	−0.734 (1.420)	−0.147	−0.300 (1.454)	−0.060
Digital home learning activities	-	-	0.193 (0.259)	0.071	-	-	0.416 (0.223)	0.166	-	-	0.540 (0.414)	0.124
*R* ^2^	0.018		0.023		0.107		0.131		0.047		0.060	
Adjusted *R*^2^	−0.022		−0.025		0.071		0.089		0.007		0.013	
Δ*R*^2^	-		0.004		-		0.025		-		0.014	
*F*	0.46		0.47		2.97 *		3.10 **		1.17		1.27	
Δ*F*	-		0.55		-		3.47	-		1.71		

Note. *N* = 129. Model 1 included child age, child sex, parent age, and parent education. Model 2 additionally included digital home learning activities. Reference categories were boy for child sex and secondary education for parent education. *B* = unstandardized coefficient; *SE* = standard error; β = standardized coefficient. * *p* < 0.05, ** *p* < 0.01.

**Table 7:** Hierarchical multiple regression analyses predicting CBCL outcomes from digital home learning activities in Group B.

	CBCL Internalizing Problems				CBCL Externalizing Problems			
	Model 1		Model 2		Model 1		Model 2	
Predictor	*B* (*SE*)	β	*B* (*SE*)	β	*B* (*SE*)	β	*B* (*SE*)	β
Child age (months)	0.303 (0.825)	0.029	0.232 (0.820)	0.022	3.869 * (1.615)	0.183	3.784 * (1.615)	0.179
Child sex (0 = boy, 1 = girl)	−0.385 (0.494)	−0.120	−0.460 (0.492)	−0.144	−1.851 (0.967)	−0.289	−1.940 * (0.969)	−0.303
Parent age (years)	−0.059 (0.051)	−0.089	−0.054 (0.051)	−0.081	0.182 (0.100)	0.136	0.188 (0.100)	0.141
Parent education (bachelor/associate vs. secondary education)	−0.930 (0.740)	−0.291	−0.924 (0.735)	−0.289	0.489 (1.449)	0.076	0.496 (1.449)	0.077
Parent education (postgraduate vs. secondary education)	−1.369 (0.820)	−0.428	−1.224 (0.819)	−0.383	−1.430 (1.606)	−0.223	−1.257 (1.613)	−0.196
Digital home learning activities	-	-	0.500 (0.276)	0.139	-	-	0.593 (0.544)	0.083
*R* ^2^	0.029		0.048		0.071		0.078	
Adjusted *R*^2^	0.001		0.014		0.044		0.045	
Δ*R*^2^	-		0.019		-		0.007	
*F*	1.02		1.41		2.58 *		2.35 *	
Δ*F*	-		3.27		-		1.19	

Note. *N* = 174. Model 1 included child age, child sex, parent age, and parent education. Model 2 additionally included digital home learning activities. Reference categories were boy for child sex and secondary education for parent education. *B* = unstandardized coefficient; *SE* = standard error; β = standardized coefficient. * *p* < 0.05.

**Table 8:** Hierarchical multiple regression analysis predicting digital home learning activities in the combined sample.

	Model 1		Model 2	
Predictor	*Β* (*SE*)	β	*Β* (*SE*)	β
Group (B vs. A)	−0.900 (0.116) ***	−0.819	−0.840 (0.116) ***	−0.764
Child age (months)			0.097 (0.095)	0.053
Child sex (0 = boy, 1 = girl)			−0.107 (0.114)	−0.098
Parent age (years)			−0.028 (0.011) *	−0.134
Parent education (bachelor/associate vs. secondary education)			−0.034 (0.151)	−0.031
Parent education (postgraduate vs. secondary education)			−0.447 (0.179) *	−0.407
*R* ^2^	0.165		0.214	
Adjusted *R*^2^	0.162		0.198	
Δ*R*^2^	-		0.048	
*F*	60.30 ***		13.60 ***	
Δ*F*	-		3.69 **	

Note. *N* = 307. Model 1 included group only. Model 2 additionally included child age, child sex, parent age, and parent education. Reference categories were Group A for group, boy for child sex, and secondary education for parent education. *B* = unstandardized coefficient; *SE* = standard error; β = standardized coefficient. * *p* < 0.05, ** *p* < 0.01, *** *p* < 0.001.

## Data Availability

The data presented in this study are available on request from the corresponding author due to ethical and privacy restrictions. The dataset contains participant-level data reported by parents for young children and is therefore not publicly available.
